# Are blood group isoantigens lost from malignant prostatic epithelium? Immunohistochemical support for the preservation of the H isoantigen.

**DOI:** 10.1038/bjc.1986.53

**Published:** 1986-03

**Authors:** P. Vowden, A. D. Lowe, E. S. Lennox, N. M. Bleehen

## Abstract

**Images:**


*Br. J. Cancer* (1986), **53**, 307–312

### Are blood group isoantigens lost from malignant prostatic epithelium? Immunohistochemical support for the preservation of the H isoantigen

P. Vowden[1]*, A.D. Lowe[2], E.S. Lennox[2] & N.M. Bleehen[1]

[1]*MRC Clinical Oncology and Radiotheraputics Unit;* [2]*The Laboratory of Molecular Biology, MRC Centre, Cambridge, UK.*

**Summary** Previous studies while demonstrating the presence of blood group isoantigens on normal prostatic epithelium have failed to identify such antigens on malignant prostatic tissue. Using a series of blood group specific monoclonal antibodies directed towards the A, B, H and Y antigens we have reinvestigated blood group isoantigen expression in both benign prostatic hypertrophy and prostatic adenocarcinoma. Results obtained from areas of benign prostatic hypertrophy are in broad agreement with those published however though we were unable to detect either A or B blood group isoantigens Type 2H and Y isoantigens were identified in 10 of the 12 tumours. These findings, while differing from previously reported results, lend support to the suggested connection between ontogenesis, oncogenesis and blood group isoantigen expression and also support the proposed link between Type 2 structures and malignant transformation.

The pattern of epithelial A, B and H isoantigen expression during both foetal development and adult life has been described by Szulman (1960, 1962, 1964). These studies have suggested that a relationship exists between blood group isoantigen (BGI) expression and ontogenesis. Additional support relating the progressive development of BGI to the degree of cellular differentiation has come from other studies. Szulman (1969, 1962) observed that while BGIs were absent from the germinal layer of epithelial structures such as cervical epithelium, they gained expression. In a study on cervical derived HeLa related cellular differentiation to changes in BGI expression. In a study on cervical derived HeLa cells (Pann & Kuhns, 1972) a similar relationship was identified between a BGI negative (H−) germinative cell layer and a BGI positive (H+) exfoliated cell population which developed after several days in culture.

Further evidence linking blood group carbohydrate chain development to both ontogenesis and oncogenesis has arisen from the examination of more complex carbohydrate structures conferring either the A or H isoantigens to a cell. Hakomori and colleagues, by identifying and detailing the distribution of the blood group carbohydrate structures ($A^a, A^b, A^c, A^d$ and $H_1, H_2$ and $H_3$ antigens) in foetal and adult erythrocytes and in gastrointestinal tumours, established the general concept that: (a) ontogenesis is accompanied by a step-by-step elongation and arborization of a complex carbohydrate chain, and (b) blocking the process of elongation and arborization occurs during oncogenesis as a result of a blocked ontogenic programme (Watabane & Hakomori, 1976; Hakomori *et al.*, 1977).

A relationship between BGI expression and malignant transformation has now been well established in a number of epithelial malignancies (Finan *et al.*, 1983; Thorpe *et al.*, 1983). Several mechanisms for the change in BGI expression that occurs have been postulated. It would seem most likely that the well documented reduction in blood group structures that occur in the majority of epithelial tissues following the development of malignancy results from the progressive loss of the necessary glycosyl transferases. A reduction in the level of these enzymes from those seen in normal tissue has been noted in material derived from several malignancies (Stellner *et al.*, 1973; Kim & Isaacs, 1975). Such an interference with the progressive development of BGIs would be expected to be associated with an accumulation of blood group precursor substances, the deletion of A and B glycosyl transferases being associated with an accumulation of H substance. It has been suggested that a Type 2 chain carbohydrate structure is the most prevalent form of A, B and H antigen present in malignant tissue (Wherrett & Hakomori, 1973) it would thus be expected that the Type 2H isoantigen and its difucosyl substituted counterpart, the Y antigen would be the dominant blood group substances in malignant tissue.

Correspondence: P. Vowden.
*Present address: St James University Hospital, Leeds, UK.
Received 23 August 1985; and in revised form, 5 November 1985.

© The Macmillan Press Ltd., 1986

Previous studies on BGI expression in normal, benign and malignant prostatic tissue have produced conflicting results. In Szulman's original studies (1960) on A and B BGIs in adult tissue it was reported that specific staining was seen at the luminal tips of single or groups of glandular epithelial cells in secretor subjects only while in areas of benign hypertrophy and corposa amylacea no BGIs were identified. Gupta *et al.* (1973) using a mixed cell agglutination reaction and formalin-fixed paraffin-embedded material described an increase in BGIs in the majority of cases of benign prostatic hypetrophy while in primary and metastatic carcinoma they found no BGIs.

This report describes the results of a study employing a battery of McAbs to determine the extent of BGI expression within benign prostatic hypertrophy and malignancy of the prostate. The aim being to investigate the possibility that Type 2H and Y isoantigens are expressed on malignant epithelium while A and B antigens are lost.

### Materials and methods

### *Histological material*

We have carried out two studies on the effects of tissue processing on BGI expression. Using specimens from colon, stomach, pancreas and breast we have established that although paraffin processing reduces the available BGI it never converted an antigen positive cryostat section to an antigen deficient paraffin section. In addition we examined the effects of each stage of the fixing and paraffin-embedding process on BGI expression by HT29 cells. (A group A human colon cell line that expresses A, H and Y antigens in culture.) These studies demonstrated that a reduction in BGI expression occurred during tissue processing. However, although individual cells become 'antigen deficient' the overall staining of each cell clump remained positive (unpublished data). For ease of access to material we therefore used formalin-fixed paraffin-embedded specimens obtained from the Pathology Department, Addenbrooke's Hospital, Cambridge, England for this study. A minimum of three blocks containing prostatic chippings removed during transurethral prostatic resection from 12 cases of benign prostatic hypertrophy and 12 cases of prostatic malignancy were examined. The distribution of blood groups is shown in the accompanying tables. In addition 6 specimens of normal prostatic tissue (2 group A, 1 group B and 3 group O) were examined.

### *Monoclonal antibodies*

Six mouse McAbs with known blood group specificity were used.

*Anti-A and Anti-B* Supernatant from stable cloned hybrids secreting mouse anti-A (A15/3D3.92.1) and anti-B (NB1/19.112.28) McAbs were obtained from the MRC Laboratory of Molecular Biology, Cambridge. Both are IgM type immunoglobulins, the supernatants of each containing $\sim 14\,\mu g\,ml^{-1}$ of immunoglobulin. The specificities of these McAbs and their use as immunohistochemical reagents have been described elsewhere (Voak *et al.*, 1982; Lowe *et al.*, 1983; Finan *et al.*, 1983; Chapman *et al.*, 1983).

*Anti-H* Three anti-H McAbs were used in this study. Two, 101 McAb and 102 McAb, were kindly provided by Dr Pastan (Laboratory of Molecular Biology, National Institute of Health, National Cancer Institute, Bethesda, Maryland 20205, USA). They are both the product of a hybridoma obtained by immunizing mice with a human epidermoid carcinoma cell line A431. McAb 101 was found to precipitate a receptor for epidermal growth factor (EGF) and also to bind to several neutral glycolipids. Binding inhibition assays initially suggested that this antibody was directed against human blood group H Type 1 sugar sequence Fuc$\alpha$(1–2)Gal$\beta$(1–3)GlcNAc..., a sequence which presumably also occurs in the EGF receptor glycoprotein (Fredman *et al.*, 1983; Richert *et al.*, 1983). Binding assays have recently shown that 101 McAb does in fact bind to both Type 1 and 2 H structures (Dr Pastan personal communication). 102 McAb is the product of another hybridoma obtained from the same fusion experiment. Binding inhibition assays have however shown that this McAb is directed against human blood group H Type 2 structure Fuc$\alpha$(1–2)Gal$\beta$(1–4)GlcNAc... (Fredman *et al.*, 1983). Both these McAbs were provided as samples of ascitic fluid. 101 McAb is an IgG class antibody, the ascitic fluid containing $500\,\mu g\,ml^{-1}$ of immunoglobulin, 102 McAb is IgM class, the ascitic fluid containing $48\,\mu g\,ml^{-1}$ of immunoglobulin. Both McAbs were used at a dilution of 1:75 in PBS (Dulbecco's 'A' tabs: Oxoid Ltd., Basingstoke, UK). An additional comparison was made with a commercial anti-H mouse McAb (Dako Corporation, USA). This McAb was used at the suggested dilution of 1 in 20.

*Anti-Y* F-3 McAb was kindly provided by Dr K.O. Lloyd (Memorial Sloan-Kettering Cancer Centre, New York, NY 10021) and is the product of a hybrid clone resulting from a fusion using spleen cells from a mouse immunised with a human lung cancer cell line (SK-LC-3). Binding and inhibition studies with a series of glycolipids with H and Lewis specificities and both Type 1 and Type 2 backbone structure have established that the only antigen to which binding occurs is the difucosyl Type 2 structure, the Y antigen (Lloyd *et al.*, 1983). In this study a sample of ascitic fluid obtained from Dr Lloyd was diluted 1:150 in PBS.

Optimal dilutions were established for all McAbs and were defined as the concentration that produced the maximum staining of endothelial and red cell elements with acceptable (or absent) non-specific background staining. All McAbs contained 0.1% azide and were stored at −20°C. Those samples in current use were kept at 4°C.

### *Immunoperoxidase technique*

The use of McAbs in an indirect immunoperoxidase technique has been described elsewhere (Finan *et al.*, 1982*a*, *b*). Briefly 5 μm sections were cut from each paraffin block and mounted on microscope slides previously treated with a chrome alum solution. Sections were then dewaxed in xylene and rehydrated through alcohols to water. Endogenous peroxidase activity was blocked by incubating the slides with hydrogen peroxide and non-specific binding blocked with rabbit serum. Sections were then incubated for 30 min with 100 μl of either a blood group specific McAb or a control solution. After further washes in PBS the sections were then incubated with 100 μl of a 1:100 dilution, in PBS, of rabbit anti-mouse peroxidase conjugate (Miles-Yeda Ltd., Rehovot, Israel). After further washes in PBS sections were flooded with a freshly prepared solution containing 10 mg diaminobenzidine, 40 μl 100 vol hydrogen peroxide and 20 ml PBS for 5 min. Slides were then counterstained with Mayer's haemalum, blued, dehydrated, cleared and mounted in DPX mountant. Sections were viewed under an Olympus CH microscope and photographed onto KB14 film (ASA 20).

### *Controls*

Positive controls were provided by sections of gastric mucosa from a group AB patient. Vascular endothelium and erythrocytes present in tissue sections acted as internal positive and negative controls for each specimen. Medium controls included incubation with inappropriate blood group McAbs, antibody free medium (Dulbecco's Modified Eagles essential medium with 10% foetal calf serum), PBS, non-immune mouse serum diluted 1:150 in PBS and absorbed McAbs produced by incubating the appropriate McAb overnight with isologous erythrocytes at 4°C.

**Table I** Staining observed in the 12 specimens of benign prostatic hypertrophy

| | *Monoclonal antibodies* | | | | | |
|---|---|---|---|---|---|---|
| *Blood group* | *A15/3D3* | *NB1/19* | *Dako H* | *101* | *102* | *F-3* |
| Group A 4 | 4 | 0 | 4 | 4 | 4 | 4 |
| Group B 2 | 0 | 2 | 2 | 2 | 2 | 2 |
| Group O 6 | 0 | 0 | 6 | 6 | 6 | 6 |

Numbers indicate those specimens showing staining with each McAb.

### Results

In areas of normal prostatic tissue little difference in staining intensity was noted when either the appropriate anti-A or anti-B McAbs were used or when any of the three anti-H or the anti-Y McAbs were used. Generally areas of benign prostatic hypertrophy showed less staining than normal structures. A considerable variation in staining intensity between apparently histologically similar areas within the same specimen was a common finding. A, B and H isoantigens were all found within the appropriate specimens (see Table I). Figure 1 shows the typical pattern of antigen expression seen in specimens showing benign prostatic hypertrophy.

Within malignant prostatic epithelium a different pattern of staining was seen. We failed to identify either A (0 from 5 group A specimens) or B (0 from 1 group B specimen) BGIs while the H and Y isoantigens were identified in 10 of 12 specimens (see Table II). Figure 2 shows the pattern of staining seen in a prostatic tumour from a group A specimen stained with 102 McAb. Of the anti-H McAbs studied 102 McAb detected a Type 2H structure in all 10 antigen positive tumours (the 5 group A and the group B tumours all displaying a Type 2H antigen). The remaining anti-H McAbs produced staining in only 3 of the 12 tumours (101 McAb) and 1 of the 12 tumours (commercial anti-H) respectively. Areas of normal prostatic epithelium present in the same 12 specimens stained with all three anti-H McAbs. Staining with F-3 (anti-Y) McAb almost exactly paralleled that seen with McAb 102, the Y antigen being detected in 10 of 12 tumours. However, in 2 cases staining could only be detected with either 102 or F-3 McAb.

Staining was noted in the positive control gastric mucosa slides with all six McAbs. Similarly vascular structures and erythrocytes stained with the appropriate McAbs in all cases. At the dilutions used however this was much more marked with A15/3D3.92.1 and NB1/19.112.28 than with the anti-H and Y McAbs. None of the negative control slides showed staining of erythrocytes, endothelium or epithelial structures, in addition no inappropriate

**Figure 1** Section from a group A specimen showing benign prostatic hypertrophy. Weak staining of isolated epithelial cells and staining of blood vessels with A15/3D3.92.1 McAb. ×200. Counterstained with haemalum.

**Figure 2** Section from a group A moderately well differentiated prostatic adenocarcinoma stained with 102 McAb. Note the widespread but patchy intensity of staining seen in this tumour which failed to stain for the A isoantigen. ×2.. Counterstained with haemalum.

BGIs were identified. Where seen, normal urothelium was found to express the appropriate BGI in all cases.

### Discussion

In this study on BGI expression in prostatic epithelium we have found a pattern of BGI expression in normal tissue that closely agrees with that described by Szulman (1960, 1962, 1964) but which differs somewhat from those observations made by Gupta *et al.* (1973) who found an increase in BGIs in benign prostatic hypertrophy. In the present study no such increase was found.

In the areas of malignant transformation marked differences exist between our observations and those reported previously. Gupta *et al.* (1973), using a mixed cell agglutination reaction, A and B antisera and Ulex europus extract (anti-H) failed to identify BGIs in 15 cases of primary and secondary adenocarcinomas of the prostate. While we failed to demonstrate the presence of either A or B isoantigens, both H and Y isoantigens were readily demonstrated in 10 of 12 (84%) of the primary prostatic adenocarcinomas examined. Maximal staining of malignant tissue was observed with 102 and F-3 McAbs both of which have been demonstrated to bind specifically to Type 2 structures. These findings may therefore give some support to suggestions made by Wherrett and Hakomori (1973) that Type 2 structures predominante in malignant epithelium. It would seem more likely however that both Type 1 and 2 structures are present in malignant tissue for both Hansson *et al.* (1983) and Picard and Feizi (1984) have demonstrated that antigens with a Type 1

**Table II** Distribution of staining observed in the 12 prostatic tumours examined

| | | *Monoclonal antibodies* | | | | | |
|---|---|---|---|---|---|---|---|
| *Histology* | *Blood group* | *A15/3D3* | *NB1/19* | *Dako H* | *101* | *102* | *F-3* |
| Well Dif. | A | – | – | + | – | ++ | ++ |
| Well Dif. | B | – | – | – | – | + | ++ |
| Well Dif. | O | – | – | – | – | – | + |
| Well Dif. | O | – | – | – | – | – | – |
| Mod. W. Dif. | O | – | – | +– | +– | ++ | ++ |
| Mod. W. Dif. | O | – | – | – | – | ++ | ++ |
| Mod. W. Dif. | O | – | – | + | + | ++ | +++ |
| Mod. Dif. | A | – | – | + | +– | +/++ | +/++ |
| Mod. Dif. | A | – | – | – | – | +/– | – |
| Mod. Dif. | A | – | – | +– | –/++ | ++ | +++ |
| Poorly Dif. | A | – | – | – | – | + | + |
| Poorly Dif. | O | – | – | – | – | –/++ | –/++ |

Staining intensity has been graded between negative (–) and +++, where considerable variation exists within a tumour the two extremes have been shown (/), +– indicates that only isolated cells showed BGI expression.

structure are present in gastrointestinal tract malignancies.

Differences in the relative affinity of Ulex europus extracts for Type 1 and Type 2 structures have been demonstrated but as preferential binding is to a Type 2H antigen the difference between our results and those reported earlier cannot be explained on these grounds. A number of previous studies have however indicated that Ulex europus extract produces inconsistent results when used to localise the H isoantigen (Cooper & Haesler, 1978; Ramsey, 1980). Our use of anti-H McAbs probably explains the increase in H isoantigen detection in the present series.

The staining of malignant prostatic tissue with F-3 McAb, which recognises a difucosyl Type 2 structure, the Y antigen, not only offers support for the presence of a Type 2H structure within the adenocarcinomas examined but also may be taken as indirect evidence for a reduction in the levels of A and B glycosyl transferases. In studies of specific glycosyl transferases in malignant tissue both Stellner *et al.* (1973) and Kim & Isaacs (1975) have established that the enzymes necessary to confer either A or B characterisation to the stem structure, the H antigen, are often reduced in malignant cells. The present study would certainly offer support for these findings.

To conclude, in contrast to previous reports results we have demonstrated the presence of BGI in prostatic malignancies, It would appear that both A and B structures are lost or greatly reduced in the malignant process but that there is a continued expression of both the H and Y antigens in the great majority of these tumours. The staining observed indicates that a Type 2H structure is maintained and may well predominate in malignant tissue within the prostate. Further studies are being undertaken to determine whether this pattern persists in other malignancies.

### References

CHAPMAN, C.M., ALLHOFF, E.P., PROPPE, K.H. & PROUT, G.R. (1983). Use of monoclonal antibodies for the localization of tissue isoantigens A and B in transitional cell carcinoma of the upper urinary tract. *J. Histochem. Cytochem.*, **31**, 557.

COOPER, H.S. & HAESLER, W.E. (1978). Blood group substances as tumour antigens in the distal colon. *Am. J. Clin. Path.*, **69**, 594.

FINAN, P.J., ANDERSON, J.R., DOYLE, P.T., LENNOX, E.S. & BLEEHEN, N.M. (1982*a*). The prediction of invasive potential in superficial transitional cell carcinoma of the bladder. *Br. J. Urology*, **54**, 720.

FINAN, P.J., GRANT, R.M., DE MATTOS, C., TAKAI, F., BERRY, P.J., LENNOX, E.S. & BLEEHEN, N.M. (1982*b*). The use of immunohistochemical techniques as an aid in the early screening of monoclonal antibodies to human colonic epithelium. *Br. J. Cancer.*, **46**, 9.

FINAN, P.J., WRIGHT, D.G.D., LENNOX, E.S., SACKS, S.H. & BLEEHEN, N.M. (1983). Human blood group isoantigen expression on normal and malignant gastric epithelium studied with anti-A and anti-B monoclonal antibodies. *J. Natl Cancer Inst.*, **70**, 679.

FREDMAN, P., RICHERT, N.D., MAGNANI, J.L., WILLINGHAM, M.C., PASTAN, I. & GINSBURG, V. (1983). A monoclonal antibody that precipitates the glycoprotein receptor for epidermal growth factor is directed against the human group H Type 1 antigen. *Fed. Proc.*, **42**, 1988 (Abstract)

GUPTA, R.K., SCHUSTER, R. & CRISTIAN, W.D. (1973). Loss of isoantigens A, B and H in the prostate. *Am. J. Path.*, **70**, 439.

HAKOMORI, S., WATANABE, K. & LAINE, R.A. (1977). Glycosphingolipids with blood group A, B, H and I activity and their changes associated with ontogenesis and oncogenesis. *Pure & Appl. Chem.*, **49**, 1215.

HANSSON, C.G., KARLSSON, K.-A., LARSON, G. & 5 Others (1983). Mouse monoclonal antibodies against human cancer cell lines with specificities for blood group and related antigens. *J. Biol. Chem.*, **258**, 4091.

KIM, Y.S. & ISAACS, R. (1975). Glycoprotein metabolism in inflammatory and neoplastic disease of the colon. *Cancer Res.*, **35**, 2092.

LLOYD, K.O., LARSON, G., STROMBERG, N., THURIN, J. & KARLSSON, K.A. (1983). Mouse monoclonal antibody F-3 recognizes the difucosyl Type 2 blood group structure. *Immunogenetics*, **17**, 537.

LOWE, A.D., LENNOX, E.S. & VOAK, D. (1983). A new monoclonal anti-A: culture supernatant with the performance of hyperimmune human reagents. *Vox. Sang.*, **46**, 29.

PANN, C. & KUHNS, W.J. (1972). Differentiation of HeLa cells with respect to group H antigen. *Nature (London)*, **240**, 22.

PICARD, J.K. & FEIZI, T. (1984). Carbohydrate antigens on glycoproteins of the neoplastic and uninvolved mucosae of patients with carcinoma of the stomach and colon. *Biochem. Soc. Transact.*, **12**, 653.

RAMSEY, E.W. (1980). Specific red cell adherence technique. *I. Urology*, **124**, 304 (letter).

RICHERT, N.D., WILLINGHAM, M.C. & PASTAN, I.H. (1983). Epidermal growth factor receptor: characterisation of a monoclonal antibody to the receptor of A431 cells. *Fed. Proc.*, **42**, 1904 (Abstract).

STELLNER, K., WATANABE, K & HAKOMORI, S. (1973). Enzymatic convertion of '$H_1$-glycolipid' to A or B-glycolipid and deficiencies of these enzyme activities in adenocarcinoma. *Biochem. Biophys. Res. Comm.*, **55**, 439.

SZULMAN, A.E. (1960). The histological distribution of blood group substances A and B in man. *J. Exp. Med.*, **111**, 785.

SZULMAN, A.E. (1962). The histological distribution of blood group antigens in man as disclosed by immunofluorescence: II. The H antigen and its relationship to A and B antigens. *J. Exp. Med.*, **115**, 977.

SZULMAN, A.E. (1964). The histological distribution of blood group antigens in man as disclosed by immunofluorescence: III. The A, B and H antigens in embryos and foetuses from 18 mm in length. *J. Exp. Med.*, **119**, 503.

THORPE, S.J., ABEL, P., SLAVIN, G. & FEIZI, T. (1983). Blood group antigens in normal and neoplastic bladder epithelium. *J. Clin. Path.*, **36**, 873.

VOAK, D., LENNOX, E.S., SACKS, S., MILSTEIN, C. & DARNBOROUGH, J. (1982). Monoclonal anti-A and anti-B: Development as a cost-effective reagent. *Med. Lab. Sci.*, **39**, 109.

WATANABE, K. & HAKOMORI, S. (1976). Status of blood group carbohydrate chains in oncogenesis and in ontogenesis. *J. Exp. Med.*, **144**, 644.

WHERRETT, J.R. & HAKOMORI, S. (1973). Characterisation of a blood group B glycolipid accumulating in the pancreas of a patient with Fabry's disease. *J. Biol. Chem.*, **248**, 3046.


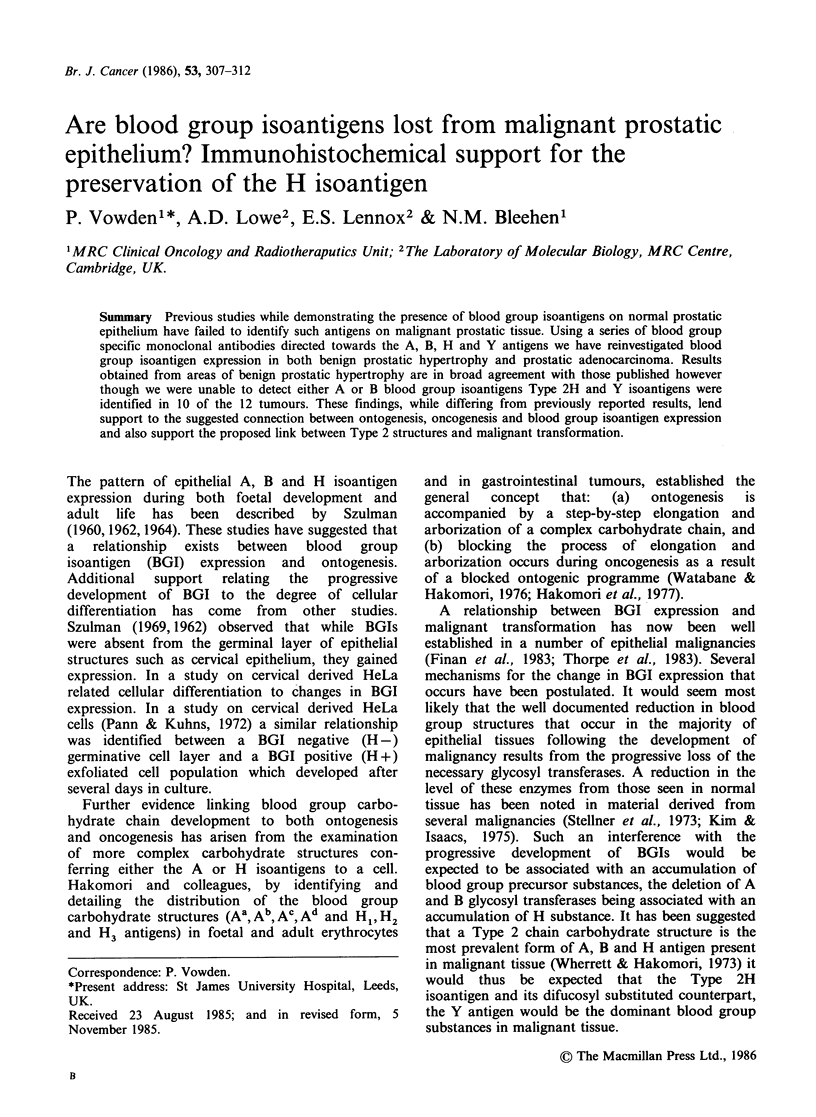

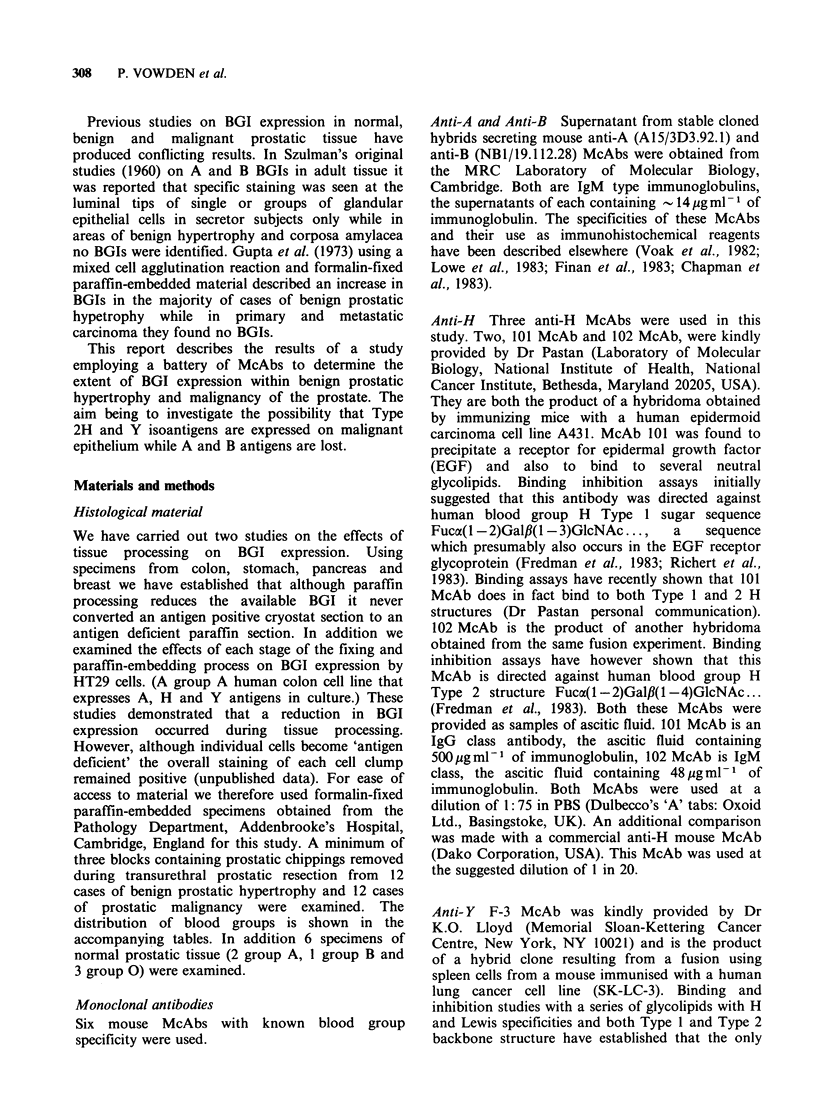

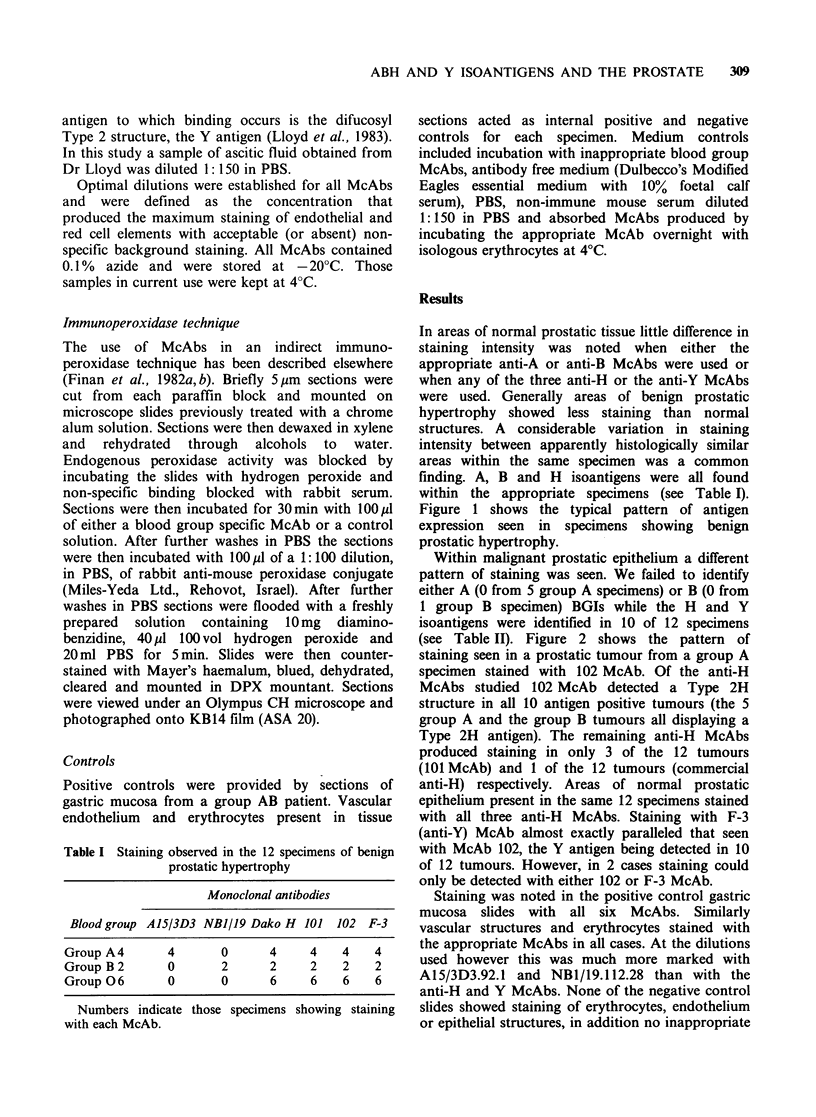

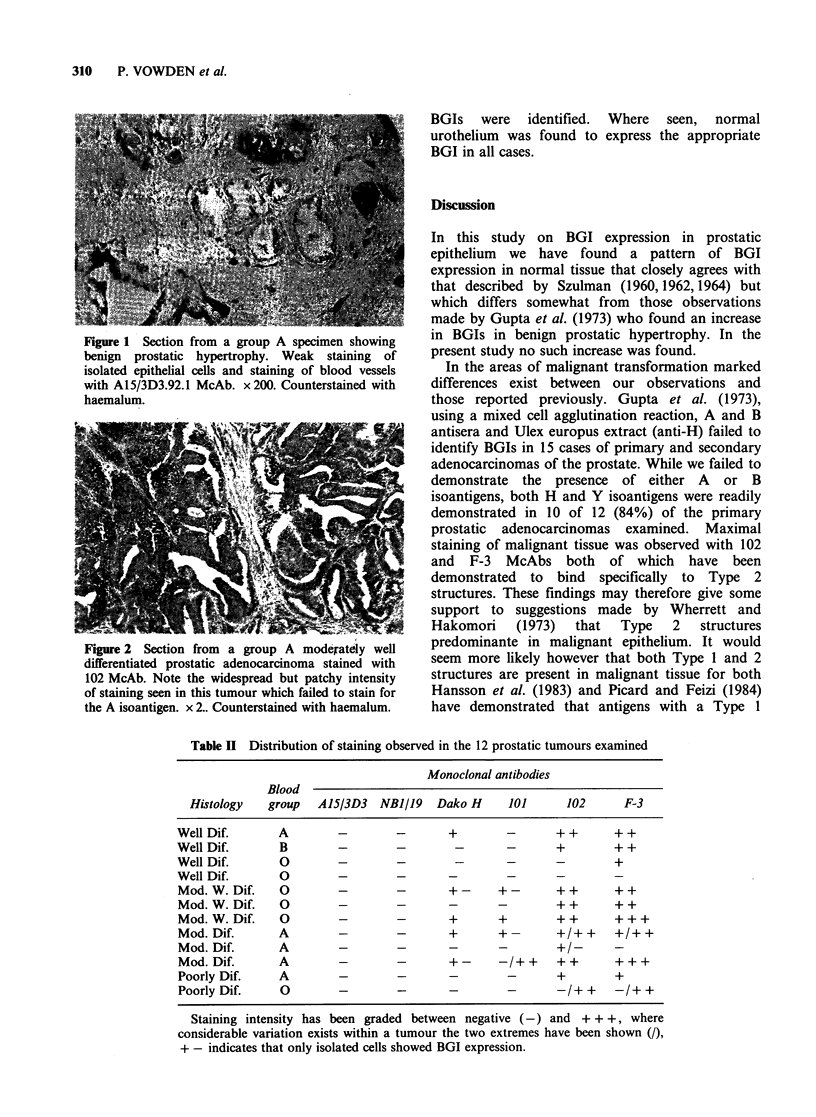

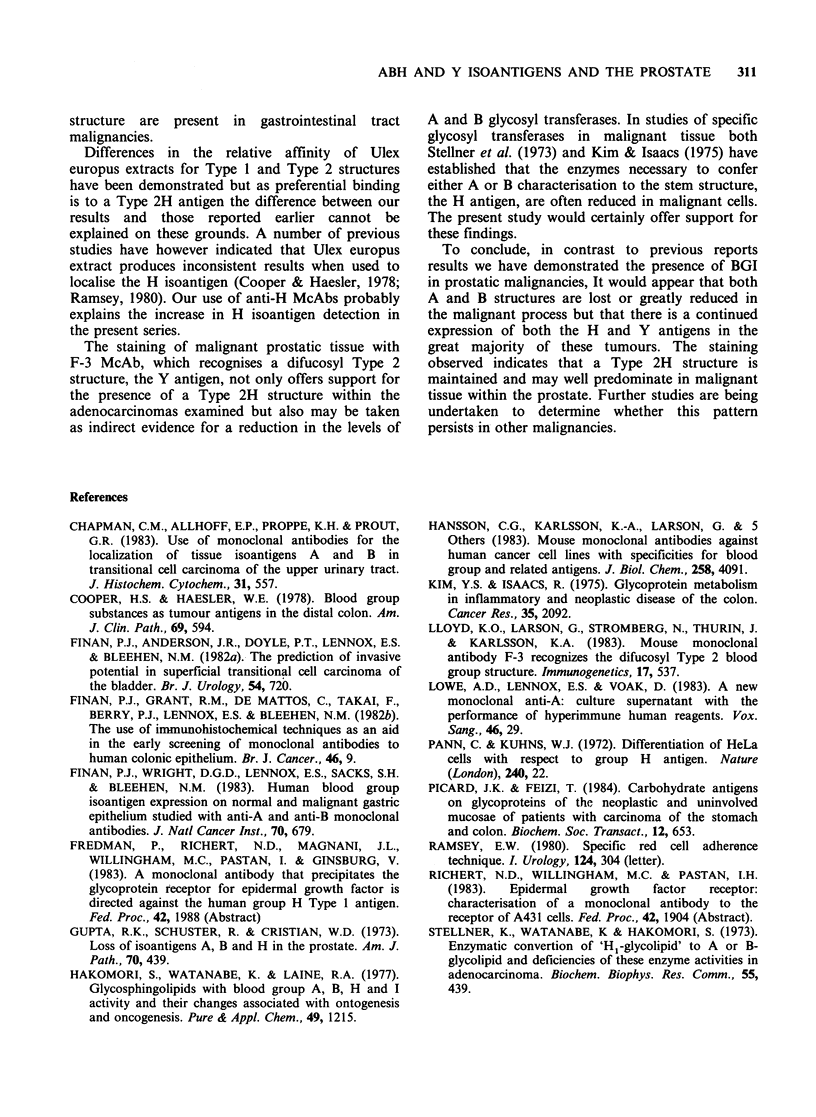

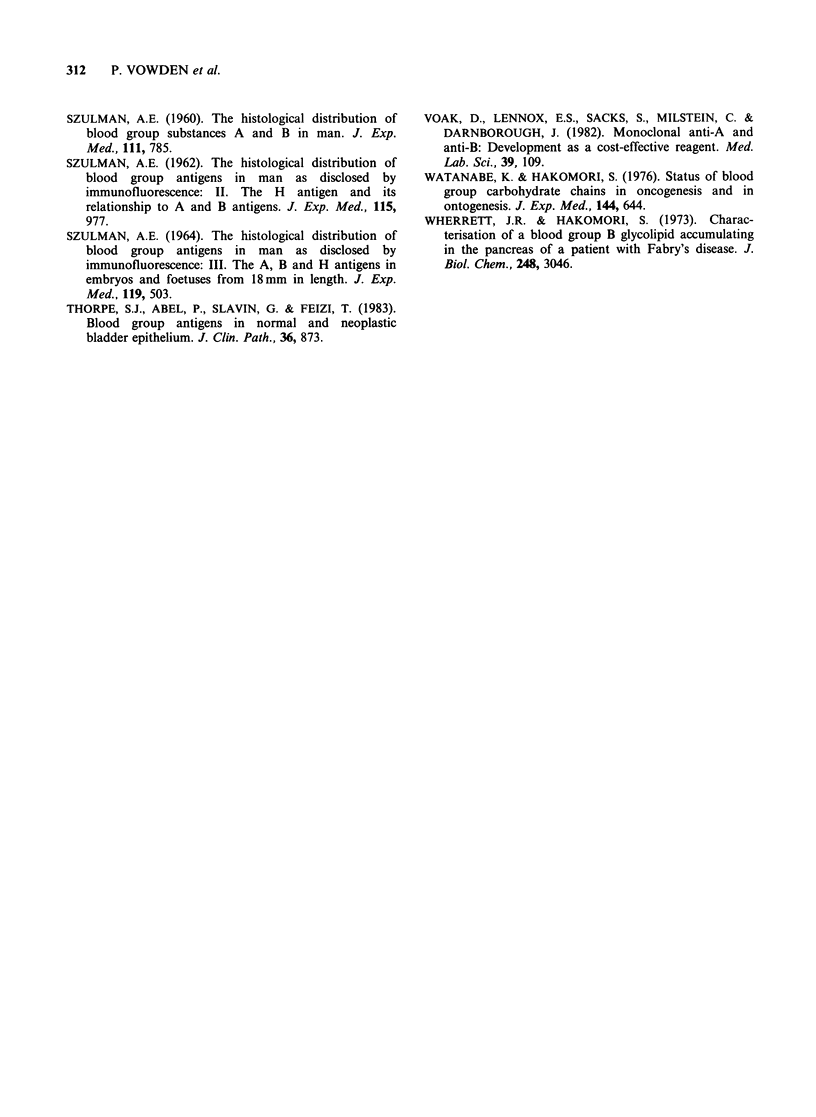


## References

[OCR_00479] Chapman C. M., Allhoff E. P., Proppe K. H., Prout G. R. (1983). Use of monoclonal antibodies for the localization of tissue isoantigens A and B in transitional cell carcinoma of the upper urinary tract.. J Histochem Cytochem.

[OCR_00486] Cooper H. S., Haesler W. E. (1978). Blood group substances as tumor antigens in the distal colon.. Am J Clin Pathol.

[OCR_00491] Finan P. J., Anderson J. R., Doyle P. T., Lennox E. S., Bleehen N. M. (1982). The prediction of invasive potential in superficial transitional cell carcinoma of the bladder.. Br J Urol.

[OCR_00497] Finan P. J., Grant R. M., de Mattos C., Takei F., Berry P. J., Lennox E. S., Bleehen N. M. (1982). Immunohistochemical techniques in the early screening of monoclonal antibodies to human colonic epithelium.. Br J Cancer.

[OCR_00504] Finan P. J., Wight D. G., Lennox E. S., Sacks S. H., Bleehen N. M. (1983). Human blood group isoantigen expression on normal and malignant gastric epithelium studied with anti-A and anti-B monoclonal antibodies.. J Natl Cancer Inst.

[OCR_00519] Gupta R. K., Schuster R., Christian W. D. (1973). Loss of isoantigens A, B and H in prostate.. Am J Pathol.

[OCR_00530] Hansson G. C., Karlsson K. A., Larson G., McKibbin J. M., Blaszczyk M., Herlyn M., Steplewski Z., Koprowski H. (1983). Mouse monoclonal antibodies against human cancer cell lines with specificities for blood group and related antigens. Characterization by antibody binding to glycosphingolipids in a chromatogram binding assay.. J Biol Chem.

[OCR_00536] Kim Y. S., Isaacs R. (1975). Glycoprotein metabolism in inflammatory and neoplastic diseases of the human colon.. Cancer Res.

[OCR_00541] Lloyd K. O., Larson G., Strömberg N., Thurin J., Karlsson K. A. (1983). Mouse monoclonal antibody F-3 recognizes the difucosyl type-2 blood group structure.. Immunogenetics.

[OCR_00547] Lowe A. D., Lennox E. S., Voak D. (1984). A new monoclonal anti-A. Culture supernatants with the performance of hyperimmune human reagents.. Vox Sang.

[OCR_00553] Pann C., Kuhns W. J. (1972). Differentiation of HeLa cells with respect to blood group H antigen.. Nat New Biol.

[OCR_00595] SZULMAN A. E. (1964). THE HISTOLOGICAL DISTRIBUTION OF THE BLOOD GROUP SUBSTANCES IN MAN AS DISCLOSED BY IMMUNOFLUORESCENCE. III. THE A, B, AND H ANTIGENS IN EMBRYOS AND FETUSES FROM 18 MM IN LENGTH.. J Exp Med.

[OCR_00583] SZULMAN A. E. (1960). The histological distribution of blood group substances A and B in man.. J Exp Med.

[OCR_00574] Stellner K., Hakomori S., Warner G. S. (1973). Enzymic conversion of "H1-glycolipid" to A or B-glycolipid and deficiency of these enzyme activities in adenocarcinoma.. Biochem Biophys Res Commun.

[OCR_00602] Thorpe S. J., Abel P., Slavin G., Feizi T. (1983). Blood group antigens in the normal and neoplastic bladder epithelium.. J Clin Pathol.

[OCR_00607] Voak D., Lennox E., Sacks S., Milstein C., Darnborough J. (1982). Monoclonal anti-A and anti-B: development as cost-effective reagents.. Med Lab Sci.

[OCR_00618] Wherrett J. R., Hakomori S. I. (1973). Characterization of a blood group B glycolipid, accumulating in the pancreas of a patient with Fabry's disease.. J Biol Chem.

